# Nephrolithiasis as a common urinary system manifestation of inflammatory bowel diseases; a clinical review and meta-analysis

**DOI:** 10.15171/jnp.2017.42

**Published:** 2017-04-12

**Authors:** Mahboube Ganji-Arjenaki, Hamid Nasri, Mahmoud Rafieian-Kopaei

**Affiliations:** ^1^Medical Plants Research Center, Shahrekord University of Medical Sciences, Shahrekord, Iran; ^2^Department of Internal Medicine, Isfahan University of Medical Sciences, Isfahan, Iran

**Keywords:** Crohn’s disease, Nephrolithiasis, Inflammatory bowel disease, IBD, Kidney stone, Ulcerative colitis

## Abstract

**Context:**

The extra-intestinal manifestations of inflammatory bowel disease (IBD) are common and involve other organs or systems for example; urinary system.

**Evidence Acquisitions:**

For this review, we used a variety of sources by searching through Web of Science, PubMed, EMBASE, Scopus and directory of open access journals (DOAJ).

**Results:**

Urinary complications may occur in up to 22% of patients and nephrolithiasis or renal/kidney stones have been suggested to be a common manifestation of disease in forms of uric acid, calcium phosphate or calcium oxalate. We performed a meta-analysis on five clinical trials and reported that correlation between IBD and formation of stone in renal system is positive and significant (Fix-effect model; CI: 95%, *P* <0.001, and randomeffect model; CI: 95%, *P* = 0.03).

**Conclusions:**

Based on the reports of the clinical trials, calcium oxalate is more prevalent in Crohn’s disease (CD) than in ulcerative colitis (UC).

**Implication for health policy/practice/research/medical education:**

In patients with IBD, ureteral obstruction, enterovesical fistulas, and urinary tract stones have the most prevalence. Ureteral obstruction, enterovesical fistulas are directly related to disease process but urinary tract stones are indirectly. Extra-intestinal urolithiasis symptoms can particularly be found in the patients with Crohn’s disease compared with UC which might be in result of steroid dependency and/or ileal involvement of CD patients. Based on the reports of the clinical trials, calcium oxalate is more prevalent in Crohn’s disease than in UC.

## 1. Background

Inflammatory bowel diseases (IBDs) are associated with extra-intestinal manifestations such as musculoskeletal, dermatologic, ophthalmologic, hematologic, cardiovascular, pulmonary, neurologic, pancreatic, hepatobiliary and genitourinary involvement. Crohn’s disease (CD) and ulcerative colitis (UC) are the main forms of IBD that differently affect the frequent rate of extra-intestinal complications (1).

Pathogenic factors of extra-intestinal manifestations remain unclear. Some organ disorders may be linked to immunologic origins, following intestinal bacterial overgrowth or side effects of therapy used to control bowel inflammation. In overall, pathogenesis of IBD is various including genetics, immunology responses or environmental factors (2,3).

Urinary complications in IBD patients have been reported in up to 22% of subjects that ureteral obstruction, enterovesical fistulas and kidney stones are the most common manifestations (2). Urinary tract complications including ileal masses or abscesses that may result in ureteric obstruction or fistulas to the bladder or urethra are the most widespread in CD compared with UC patients (4).

In a clinical trial 312 CD patients who were considered from urinary tract complications viewpoints, 77 (24.7%) had urologic symptoms, 51 (16%) influenced by simple cystitis and the subjects which acquired structural urinary tract abnormalities had most common manifestations (5,6).

This review article tries to discuss the relations of these parameters other renal manifestations of IBD, and also tries to find out the relation between IBD and urinary system stones. We reviewed and performed a meta‏-analysis on five studies of clinical trials that assessed the occurrence of renal stones in patients with IBD.

## Materials and Methods

For this review, we used a variety of sources by searching through Web of Science, PubMed, EMBASE, Scopus and directory of open access journals (DOAJ). The search was performed using combinations of the following key words and or their equivalents such as; Crohn’s disease, nephrolithiasis, inflammatory bowel disease, ulcerative colitis and kidney stone.

## Results

The relation between IBD and renal or urologic complications is presented in Table 1. As can be seen the complications are related to the disease process or might be iatrogenic which are discussed in more detail below.

**Table 1:** Renal complications of patient in inflammatory bowel disease

**Complications directly related to disease process**
Enterourinary fistulas
Ureteral obstruction
Genital involvement
**Complications indirectly related to disease process**
Nephrolithiasis
Glomerulonephritis
Amyloidosis
**Iatrogenic complications**
Medications
Aminosalicylates
Cyclosporine

## Entero-urinary fistulas

The prevalence of entero-urinary fistulas in patients of CD is 24-32% (1). After diverticulitis and cancer, CD is estimated to be the third cause of entero-vesical fistulas (EVF) with calculating 5%-17%, first cause of ileovesical fistulas and EVF in patients higher than 40 years with accounting for more than 75% but in patients >50 years old accounts for 1.8%. EVF can cause pneumaturia and fecaluria with frequencies of 38%–94% and 17%–63%, respectively, and epididymitis, hematuria, urinary tract infections, prostatitis, fever, abdominal pain and involuntary urine passage or urorrhea (7-13).

Management options for EVF are surgery and intervention of medications. Antibiotics and anti-inflammatory medications such as aminosalicylates and corticosteroids or cyclosporine and 6-mercaptopurine have been used successfully (14-18).

## Ureteral obstruction

In 50% to 73% of patients having CD and in more than 50% of patients having UC, the obstruction ureteral is not usually occurred by stones (5). Non-calculous obstruction (NCO) in CD is due to retroperitoneal inflammation or an EVF near the ureter and surgery is the treatment choice (19).

## Genital involvement

Genital involvement in IBD is uncommon and is associated with and erythema, edema, ulceration, fibrosis of vulva, scrotum penis from direct extension of underlying disease, pyoderma gangrenosum, or abscesses, phimosis, balanoposthitis, metastatic CD or granulomatous inflammation in seminal vesicles, prostate gland, and scrotum. Genital involvement may result in perineal pain, urethral strictures, urethral discharge, fever, and might parallel the activity of underlying IBD. The treatment options for this include antibiotics, systemic steroids, immunosuppression, resection of involved bowel, skin and circumcision (20-25).

## Nephrolithiasis

The nephrolithiasis prevalence in IBD patients has been suggested to be more than general, in adults the prevalence is more than children and in CD patients is higher compared with UC patients and in CD patients who have extensive surgery is the highest (26-28).

Multiple kidney stones are occurred in IBD and in CD the stones are occurred often in the right ureter and terminal ileum (7%–15%) (5,29). The stones are usually composed of calcium oxalate that result in high urinary oxalate, calcium phosphate that result in increased Ca mobilization from bone, decreased tubular resorption of Ca and uric acid that result in decreased urate solubility in an acidic, concentrated urine. On the other hand, low levels of citrate, magnesium, anti-lithogenic agents, accompanying bile salt and fat malabsorption cause stone development (30).

The risk of calcium oxalate stone is increased in patients having an intact colon, due to high absorption of sodium-bound oxalate in colon, but in patients with an ileostomy, uric acid stones are increased because of frequent dehydration (31).

Despite existence of hyperoxaluria, hypocitraturia, hypomagnesuria, hypophosphaturia, low urinary pH and low urine volume in patients with IBD, but most of them have not renal stones (32), probably due to attention to individual genetic.

## Glomerulonephritis

The occurrence of glomerulonephritis in CD and UC has been reported (33,34). The occurrence of glomerulonephritis is not association with duration of disease, but has correlation with level of inflammation in bowel and bile duct (35-37). For improving the renal function in patients having IBD with glomerulonephritis, the use of steroids is helpful (38,39).

## Amyloidosis

Moschkowitz in 1936 reported the association between secondary amyloidosis (A) and IBD for the first time (40). It has been shown in clinical studies that amyloidosis is occurred in about 1% of IBD patients. Amyloidosis is 3-fold in male subjects compared with female ones. In CD patients the prevalence is 10-fold compared with UC patients and it correlates with extra-intestinal complications (36,41-43).

The secondary amyloidosis pathogenesis has been attributed to serum amyloid-A (SAA), which is an acute phase protein with unknown function‏ (44) that expression level is increased in response to inflammation and may lead to amyloidosis. In patients with IBD amyloidosis may affect the kidney function and cause proteinuria followed by nephrotic syndrome and subsequently renal insufficiency (3,36,45,46). Finally, in CD with azathioprine and plasmapheresis, treatment of renal amyloidosis may be useful (47,48).

## Complications of medical therapy

Drugs such as corticosteroids, azathioprine, 6-mercaptopurine, metronidazole and low dose of methotrexate have little or no nephrotoxicity (49-54). Aminosalicylates and cyclosporine are the medications with potential renal toxicity.

## Aminosalicylates (5-ASA)

Sulfasalazine (5-ASA bound to sulfapyridine), mesalamine, and olsalazine are types of aminosalicylates that renal toxicities with these agents have been reported (55-58). These are associated with clinical improvement in active UC and are essential in maintaining remission. Mesalazine is arguable in CD and seems to be beneficial only in mild ileocolonic disease (59). Renal deficiency might occur in more than 1% of patients using 5-ASA, but renal clinical disturbance may occur in only 1 to 500 IBD patients (60).

Treatment with 5-ASA may cause renal toxicity that include glomerulonephritis, change in nephropathy with interstitial nephritis and nephrotic syndrome, which in these cases might be associated with nephrogenic diabetes (61,62).

## Cyclosporine

Dose of cyclosporine (CsA) is related to nephrotoxicity (63,64). Cyclosporine has a role in tenacious UC, which by inhibiting calcineurin (a calmodulin and calcium dependent serine/threonine protein phosphatase) can block the production of interferon- γ and interleukin-2 interrupting the cellular immune response (65,66).

## Meta-analysis

We analyzed the data of 5 clinical trials by evaluating the rate difference with their 95% confidence intervals. Then, the results were reported in form of Fix and random effect- models and the presence of heterogeneity across trials by using the I^2^ statistical analysis. The analysis was performed by comprehensive meta-analysis V3 software. The results of the studies are outlined in Table 2.

**Table 2:** Clinical trials studies considered in meta-analysis

**Study name**	**IBD number**	**Non-IBD number**	**Sex (F:M)**	**CD number**	**UC number**
Nightingale et al (67)	38	46	57:27	32	6
McConnell et al (68)	40	17	24:16	25	15
Ishii et al (69)	39	59	22:76	39	0
Kumar et al (70)	48	48	21:27	11	37
Parks et al (71)	133	1156	-	-	-

**Figure 1:**
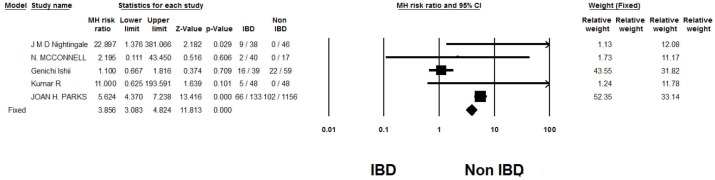
Forest plot of controlled trials of IBD and occur renal stone.

Analysis showed that correlation between IBD and renal stone is significant (Figure 1), with fix-effect model; CI: 95%, *P* value < 0.001, I^2^: 88.52 and Random-effect model; CI:95%, *P* value = 0.03.

As it was said hyperoxaluria is a common extra intestinal complication of IBD that might be result from malabsorption and the absence of intestinal oxalate degrading bacteria. Intestinal hyperoxaluria is a cause in formation of oxalate stones in kidneys. Since probiotics in treatment of IBD are efficacious, therefore some types of probiotics should be useful in reduction of renal stone in patients with IBD (72).

## Conclusion

In patients with IBD, ureteral obstruction, enterovesical fistulas, and urinary tract stones have the most prevalence. Ureteral obstruction, enterovesical fistulas are directly related to disease process but urinary tract stones are indirectly. Extra-intestinal urolithiasis symptoms can particularly be found in the patients with CD compared with UC which might be in result of steroid dependency and/or ileal involvement of CD patients. Clinical trial studies pointed that hyperoxaluria has mostly redundancy in CD that results in high urinary oxalate that would be associated with low pH and urine volume.

## Authors’ contribution

All data were independently abstracted by three authors (MGA, MRK. and HN) by using a data abstraction form (author name, sex, number of patients and controls). Analysis of tests and primary draft were preformed by MGA. The manuscript was edited by MRK and HN.

## Conflict of interest

The authors declared no competing interests.

## Funding/Support

None declared.
